# How Do Taxonomic and Functional Diversity Metrics Change Along an Aridity Gradient in a Tropical Dry Forest?

**DOI:** 10.3389/fpls.2022.923219

**Published:** 2022-07-07

**Authors:** Ana Cláudia Pereira de Oliveira, Alice Nunes, Maria Alexandra Oliveira, Renato Garcia Rodrigues, Cristina Branquinho

**Affiliations:** ^1^cE3c – Centre for Ecology, Evolution and Environmental Changes – Global Change and Sustainability Institute, Faculdade de Ciências, Universidade de Lisboa, Lisboa, Portugal; ^2^Núcleo de Ecologia e Monitoramento Ambiental, Universidade Federal do Vale do São Francisco, Petrolina, Brazil

**Keywords:** climatic gradient, dryland, functional redundancy, global change ecology, hump-shaped curve, space-for-time substitution, Caatinga

## Abstract

Ecological indicators based on biodiversity metrics are valuable and cost-effective tools to quantify, track and understand the effects of climate change on ecosystems. Studying changes in these indicators along climatic gradients in space is a common approach to infer about potential impacts of climate change over time, overcoming the limitations of lack of sufficiently long time-series data. Here, we studied the response of complementary biodiversity metrics in plants: taxonomic diversity (species richness and Simpson index) and functional diversity (diversity and redundancy) in 113 sampling sites along a spatial aridity gradient (from 0.27 to 0.69 of aridity index-AI) of 700 km in a Tropical dry forest. We found different responses of taxonomic and functional diversity metrics to aridity. Species diversity showed a hump-shaped curve peaking at intermediate levels of aridity between 0.38 and 0.52 AI as an ecotone, probably because it is where most species, from both drier and more mesic environments, still find conditions to co-exist. Functional diversity showed a positive linear relation with increasing aridity, suggesting higher aridity favors drought-adapted species with diverse functional traits. In contrast, redundancy showed a negative linear relation with increasing aridity, indicating that drier sites have few species sharing the same functional traits and resource acquisition strategies. Thus, despite the increase in functional diversity toward drier sites, these communities are less resilient since they are composed of a small number of plant species with unique functions, increasing the chances that the loss of one of such “key species” could lead to the loss of key ecosystem functions. These findings show that the integration of complementary taxonomic and functional diversity metrics, beyond the individual response of each one, is essential for reliably tracking the impacts of climate change on ecosystems. This work also provides support to the use of these biodiversity metrics as ecological indicators of the potential impact of climate change on drylands over time.

## Introduction

Anthropogenic activities, climate change, and invasive alien species have led to a global biodiversity crisis, encompassing not only biodiversity loss but also biodiversity change (Dornelas et al., 2014; Branquinho et al., 2019). These losses and changes can affect ecosystem services and consequently human well-being (Díaz et al., 2006; Cardinale et al., 2012; Mori et al., 2018). For instance, changes in plant cover can increase topsoil temperature and water evaporation, both processes associated with land degradation, which affect plant productivity and below-ground processes (Breshears et al., 1997; Manhães et al., 2022). Thus, it is urgent to identify potential ecological indicators based on biodiversity metrics (e.g., taxonomic and functional), because they integrate the effects of environmental drivers on ecosystems’ functioning accounting for ecosystems’ specificities (e.g., the same increase in temperature will afffect differently semi-arid and polar ecosystems; Branquinho et al., 2019). In addition, they respond to the need to use different diversity metrics, and test them under field conditions, to track biodiversity changes, e.g., to comply with the Convention on Biological Diversity, as tools to anticipate the integrated response of biodiversity to drivers of global change affecting ecosystems, as it has been suggested by the essential biodiversity variables (Pereira et al., 2013). This knowledge can avoid reaching tipping points (Dakos et al., 2019) and reduce mitigation and restoration costs, by implementing a proactive rather than a reactive approach (Walls, 2018).

Due to its complexity, it is extremely hard to quantify changes in the entirety of biodiversity and its properties (Lindenmayer et al., 2015) in response to drivers of change. Thus, ecological indicators are cost-effective and valuable tools that allow for summarizing a complex set of information retaining only the essential significance of the aspects being analyzed (Heink and Kowarik, 2010).

Species richness, i.e., a taxonomic metric that consists of the total number of species, has been traditionally and widely used as a proxy for biodiversity assessments (Cadotte et al., 2011). Previous studies showed that plant species richness is positively related to the ability of ecosystems in maintaining multiple functions, such as productivity and carbon storage, suggesting that conservation of plant diversity is crucial to minimize the negative effects of environmental change, particularly in drylands (Maestre et al., 2012). Yet, species richness does not consider species abundance (Magurran, 2004). The abundance and equitability of each species can be included in taxonomic metrics (Ricotta, 2005), such as the Simpson diversity index, which measures the probability of two random individuals in a community to belong to the same species (Ricotta, 2005). Additionally, taxonomic metrics consider all species and individuals as equivalents (Magurran, 2004; Cianciaruso et al., 2009), disregarding their functional role and how they affect ecosystem functioning (Naeem and Wright, 2003).

The study of species’ functional traits overcomes this limitation, providing a more mechanistic link between species and multiple ecosystem functions, such as primary productivity and nutrient cycling, as species influence these functions *via* their traits (Mason and De Bello, 2013). For instance, plant traits such as *growth form* and *leaf traits* are associated with photosynthetic production and ecophysiological adaptation e.g., to water deficit. *Height* is linked with light capture and competitive vigor. *Seed dispersal mode* influences the distance species can cover. *Spinescence* and *chemical defense* allow defense against herbivory while reducing heat or drought stress. The *trunk rhytidome* operates as a thermal insulator and barrier against excessive water loss. The *photosynthetic pathway* (C3, C4 and CAM – Crassulacean acid metabolism) describes nutrient and water use efficiency. *Roots* allow nutrient acquisition and mutualistic associations with soil micro-organisms (such as mycorrhizal fungi and nitrogen-fixing bacteria; Cornelissen et al., 2003; Hodge et al., 2009; Lewinsohn and Vasconcellos-Neto, 2009; Pérez-Harguindeguy et al., 2016). Hence, functional diversity metrics have been increasingly used to complement taxonomic metrics, as indicators of mechanisms driving changes in biological communities and as predictors of ecosystem functioning (Petchey and Gaston, 2006; Nunes et al., 2017; Sfair et al., 2018). Functional traits encompass morphological, behavioral and ecological differences among the individuals and species that can interfere with species growth, reproduction and survival (Violle et al., 2007).

Despite the success of the use of functional diversity *per se* or jointly with taxonomic metrics in ecological studies, these alone do not reflect the ability of communities to ensure the maintenance of ecosystem functions in face of environmental changes (De Bello et al., 2007). In this context, the concept of functional redundancy (FR) was proposed by Walker (1992), corresponding to a measure of how much a community is functionally saturated by different species represented by analogous traits. In this regard, FR can be used as a means of detecting the potential loss of species that carry out unique roles in important ecosystem processes as well as reorganization and renovation of the ecosystem after disturbance (resilience), with the potential to significantly affect and change ecosystem functioning (Walker, 1992; Fonseca and Ganade, 2001; De Bello et al., 2007; Pillar et al., 2013). Due to the peculiarity and, at the same time, the complementary character of these metrics, it is important to evaluate how they change along environmental gradients, and how we can interpret the observed patterns to better anticipate changes in the structure and functioning of the ecosystems to be studied over time.

Studying biodiversity changes along climatic gradients in space has become a valuable tool to understand potential changes over time due to climate change, allowing us to follow and anticipate abrupt changes in ecosystem structure and functioning. Dryland systems (composed of hyper-arid, arid, semi-arid and dry sub-humid areas) are characterized by a combination of high evaporation, low rainfall, and human activities such as livestock grazing, the collection of wood and non-wood forest products, fire use, and soil cultivation (Pennington et al., 2009). These characteristics make drylands highly vulnerable to climate and environmental changes (Maestre et al., 2012). For example, increasing aridity may cause changes in plant communities shifting from a high diversity of herbs to a few shrubs (Yao et al., 2021), as well as decrease plant functional diversity (e.g., Nunes et al., 2017) and increase FR (e.g., Le Bagousse-Pinguet et al., 2019). Additionally, global dryland areas are expected to expand due to climate change (Koutroulis, 2019). The global land surface occupied by drylands currently exceeds 47% and may increase an additional 7% by 2,100 (Koutroulis, 2019).

This study is focused on the vegetation of Caatinga, a Tropical dry forest with peculiar flora covering the semi-arid region of Brazil, and showing the highest vulnerability to climate change in that country (Sarmiento, 1975; da Silva et al., 2018). A rise in temperature ranging from 4 to 18°C (Intergovernmental Panel on Climate Change [IPCC], 2011) and a decrease in rainfall between 22 and 40% (Magrin et al., 2014; Buriti and Barbosa, 2018) are expected to occur up to 2,100 in Caatinga. Climate change, together with anthropogenic activities, will further aggravate land degradation affecting 28.6 million people highly dependent on local natural resources (da Silva et al., 2018). In this context, the main objective of this study was to find ecological indicators based on biodiversity metrics that can help track climate change effects in space to infer potential impacts of climate change over time. For this, we assessed changes in plant taxonomic and functional diversity metrics along a spatial aridity gradient of 700 km in the Caatinga ecosystem.

We hypothesized that complementary diversity metrics will respond differently to aridity in this tropical dry forest, namely that with increasing aridity we will find: (i) a decrease in species richness, only those highly adapted to drought remaining (e.g., Yao et al., 2021); (ii) a decrease in functional diversity due to environmental filtering (e.g., Nunes et al., 2017); and (iii) an increase in FR between species sharing the same drought-adapted traits (e.g., Le Bagousse-Pinguet et al., 2019).

## Materials and Methods

### Study Area

**FIGURE 1:**
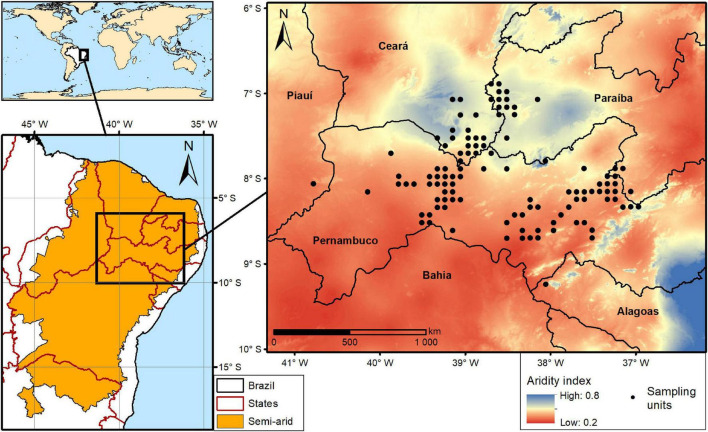
Map with the location of the study area and the 113 sampling units (black dots) distributed along a regional aridity gradient.

The present study was carried out along a regional aridity gradient of 700 km, covering four Brazilian states, namely Alagoas, Ceará, Paraíba, Pernambuco, and Piauí (Figure 1). This gradient overlaps the Caatinga Phytogeographic Domain represented by many vegetation types ranging from semi-deciduous forests to open vegetation, located in rocky outcrops in driest areas (Fernandes and de Queiroz, 2018), encompassing also local variations in land management (for more details see Oliveira et al., 2020a). The study area has a mean annual temperature of 24°C (ranging from 21 to 26°C), average annual precipitation of 680 mm (spanning from 440 to 1,098 mm), and an altitude between 278 and 930 m (Oliveira et al., 2020a). The aridity index varies from 0.27 to 0.69 including mostly semi-arid and humid climates (Oliveira et al., 2020a).

### Data Sampling

The database used in this work comprises the most abundant plant species from the Brazilian dry forest extracted from an initial database with presence records of 937 plant species, collected between 2008 and 2015. To estimate species abundances based on presence data for these 937 plant species we divided each of the 113 sampling units (10 Km × 10 Km = 11,300 Km^2^) into 25 sampling quadrats of 2 Km × 2 Km (for more details see Oliveira et al., 2020b). The sum of the presence records of each species at each small quadrat (2 Km × 2 Km) was considered a proxy of its abundance, with each species, counted only once in each quadrat (even if recorded more than once). Thus, the maximum abundance at the sampling unit level was 25. With these data we built an abundance database and selected the most dominant species comprising 80% of species relative abundance, obtaining 48 species distributed in 17 families and 42 genera. Woody and herbaceous plant species had been identified based on herbarium collections^1^, expert knowledge and specialized bibliography. Fabaceae was the most frequent family (13 species), followed by Cactaceae (eight species), Euphorbiaceae and Bromeliaceae (six species each), and Anacardiaceae (four species).

Then, we used this database to calculate taxonomic and functional diversity metrics for each sampling unit to assess the response of plant communities to climate along an aridity gradient. Taxonomic diversity was calculated as total species richness (i.e., number of different taxa found) and the Simpson diversity index (Simpson, 1949). To compute community functional metrics we selected 13 functional traits, whose individual response to aridity was addressed in a previous work (Oliveira et al., 2020a), namely: (1) growth form; (2) maximum plant height; (3) leaf phenology type; (4) leaf thickness; (5) specific leaf area; (6) root type; (7) dispersal strategy; (8) fruit type; (9) photosynthetic pathway; (10) spinescence presence; (11) presence of leaves arranged in a rosette; (12) chemical defense exudation mechanisms; and (13) rhytidome presence. These comprise continuous, categorical and binary traits reflecting plant strategies associated with plant establishment, defense, regeneration, and dispersal (Lewinsohn and Vasconcellos-Neto, 2009; Pérez-Harguindeguy et al., 2016). Trait data for the 48 most abundant species were measured directly in the field following standard protocols (for traits 1, 2, 4, 5, 10, 12, and 13), retrieved from the botanical collection of Herbarium Vale do São Francisco, Petrolina, Pernambuco, Brazil (for traits 6 and 8), or from other bibliographic sources (for traits 3, 7, 9, and 11). The categories considered for the functional classification of species in relation to categorical traits were described in a previous work (Oliveira et al., 2020a). For continuous traits, an average value *per* species was used, regardless of the plasticity of each trait, because the work was focused on the turnover between sites and not on intraspecific trait variability. We then calculated functional diversity (Rao’s quadratic entropy) and FR (De Bello et al., 2007; Pillar et al., 2013) for the plant community. Rao’s quadratic entropy (Rao’s hereinafter) may be calculated for multiple traits altogether, and it is influenced by species abundance and diversity in their traits (Botta-Dukát, 2005). Thus, its value may decrease if species richness increases, because the inclusion of a new species into the community increases the species-abundance based diversity, while it may decrease the average dissimilarity among species (Botta-Dukát, 2005). All calculations were performed with the statistical software R (The R Core Team, 2018), using the dbFD function of the FD package (Laliberté et al., 2014). FR, a feature related to the stability, resistance and resilience of ecosystems to environmental changes (Hooper et al., 2005; Guillemot et al., 2011), was also determined. FR was obtained for each sampling unit through the differences between taxonomic diversity (using the Simpson diversity index) and functional diversity (using Rao’s quadratic entropy; De Bello et al., 2007; Pillar et al., 2013).

To characterize the climatic gradient, we used the aridity index adopted by the United Nations, whose values were retrieved from a global database (Trabucco et al., 2008). The aridity index is calculated as the ratio between mean annual precipitation and annual potential evapotranspiration. Thus, lower values correspond to more arid environments, and *vice-versa*.

### Data Analysis

To evaluate the response of the plant community to aridity, we used the aridity index as a predictor to explain changes in community taxonomic and functional metrics (species richness, Simpson diversity index, Rao’s, and FR). The relationships between the aridity index and taxonomic and functional metrics were tested using general linear models, except for species richness (counts), which was analyzed using generalized linear models with Poisson distribution, accounting for overdispersion. For all models we included and tested a quadratic term for aridity, as the response of the plant community to aridity is not necessarily linear. Models’ assumptions were graphically inspected. All statistical analyses were performed using R software version 3.4 (The R Core Team, 2018).

## Results

**FIGURE 2:**
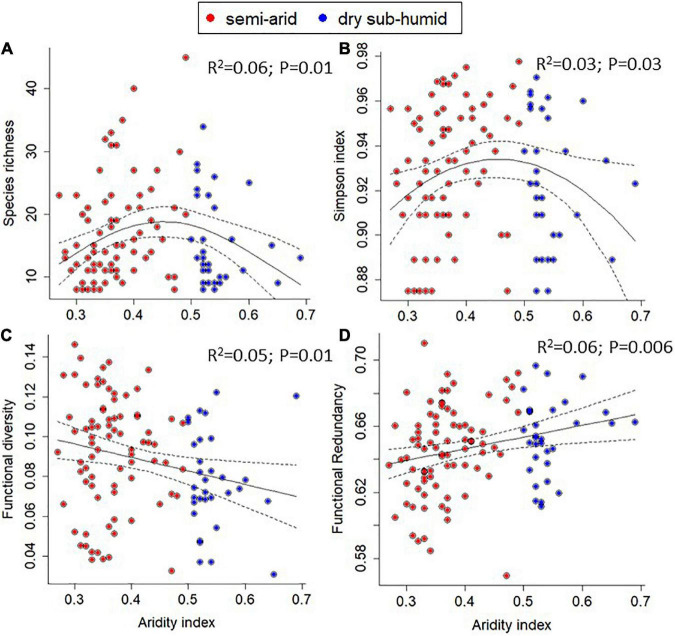
Relationships between the aridity index and: **(A)** species richness; **(B)** Simpson diversity index; **(C)** functional diversity; and **(D)** functional redundancy. Solid and dashed lines represent the fitted linear or quadratic regression and 95% confidence intervals, respectively. Adjusted *R*^2^ and associated *p*-values are also shown.

Within the 113 sampling units distributed along the aridity gradient, species richness ranged between 8 and 45 plant species (minimum and maximum values *per* sampling unit, respectively) of a maximum of 48 species (Figure 2A). Simpson diversity index ranged from 0.87 to 0.98 (Figure 2B). Functional diversity, represented by Rao’s, ranged from 0.03 to 0.15 and FR spanned from 0.57 to 0.71 (Figures 2C,D, respectively).

**FIGURE 3:**
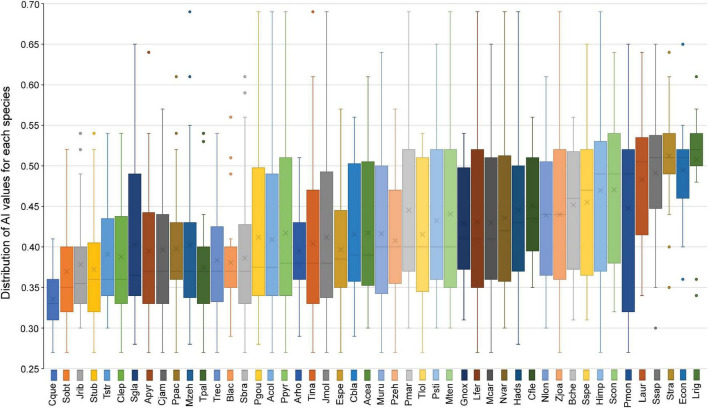
Boxplots showing the minimum, lower quartile, median, upper quartile and the maximum values of the aridity index of the sampling units where the presence of each of the dominant species (48 species) was recorded. Species are arranged in ascending order of the median of the aridity index values found along their distribution range. Abbreviated species’ names from left to right in the Figure: Cque (*Cnidoscolus quercifolius*), Sobt (*Sideroxylon obtusifolium*), Jrib (*Jatropha ribifolia*), Stub (*Spondias tuberosa*), Tstr (*Tillandsia streptocarpa*), Clep (*Commiphora leptophloeos*), Sgla (*Sapium glandulosum*), Apyr (*Aspidosperma pyrifolium*), Cjam (*Cereus jamacaru*), Ppac (*Pilosocereus pachycladus*), Mzeh (*Melocactus zehntneri*), Tpal (*Tacinga palmadora*), Trec (*Tillandsia recurvata*), Blac (*Bromelia laciniosa*), Sbra (*Schinopsis brasiliensis*), Pgou (*Xiquexique gounellei* subsp. *gounellei*), Acol (*Anadenanthera colubrina*), Ppyr (*Cenostigma pyramidale* var. *pyramidale*), Arho (*Arrojadoa rhodantha*), Tina (*Tacinga inamoena*), Jmol (*Jatropha mollissima*), Espe (*Encholirium spectabile*), Cbla (*Croton blanchetianus*), Acea (*Amburana cearensis*), Muru (*Astronium urundeuva*), Pzeh (*Parapiptadenia zehntneri*), Pmar (*Pseudobombax marginatum*), Tlol (*Tillandsia loliacea*), Psti (*Piptadenia stipulacea*), Mten (*Mimosa tenuiflora*), Gnox (*Guapira noxia*), Lfer (*Libidibia ferrea*), Mcar (*Manihot carthagenensis*), Nvar (*Neoglaziovia variegata*), Hads (*Harrisia adscendens*), Cfle (*Cynophalla flexuosa*), Nlon (*Neocalyptrocalyx longifolium*), Zjoa (*Sarcomphalus joazeiro*), Bche (*Bauhinia cheilantha*), Sspe (*Senna spectabilis*), Himp (*Handroanthus impetiginosus*), Scon (*Selaginella convoluta*), Pmon (*Pityrocarpa moniliformis*), Laur (*Luetzelburgia auriculata*), Ssap (*Sapindus saponaria*), Stra (*Senna trachypus*), Econ (*Enterolobium contortisiliquum*), and Lrig (*Microdesmia rigida*).

Taxonomic metrics, namely species richness and the Simpson diversity index, showed a significant hump-shaped relationship with the aridity index (lower value of the aridity index corresponds to higher aridity, and *vice-versa*), peaking at intermediate aridity levels, despite the considerable dispersion of values between sites (Figures 2A,B). This is supported by the best fit of the quadratic regression between taxonomic metrics and the predictor variable (Figures 2A,B and Supplementary Table 1). Plant communities with a larger number of species were found within an aridity index ranging from 0.34 to 0.52. Most species showed a widespread distribution along the studied gradient (Figure 3). Despite many species are present along the gradient [e.g., *Anadenanthera colubrina* (Vell.) Brenan, Fabaceae], although, with different abundances, there are also species of more restricted distribution, associated mainly with the more arid places (e.g., *Cnidosculus quercifolius* Pohl, Euphorbiaceae) and others to less arid ones [e.g., *Microdesmia rigida* (Benth.) Sothers & Prance, and Chrysobalanaceae; Figure 3).

In contrast with taxonomic metrics, functional metrics, namely functional diversity and FR, showed a linear trend along the aridity gradient (Figures 2C,D, respectively; Supplementary Table 1). Functional diversity increased toward more arid sites, displaying its highest values within an aridity index below 0.4 (Figure 2C). FR showed the opposite trend, increasing toward less arid conditions (Figure 2D).

## Discussion

In this study, we found different responses of taxonomic and functional diversity to the aridity gradient, highlighting the importance of using these complementary diversity metrics as ecological indicators to better understand the response of the plant community to changes in climate in space, as a potential proxy of changes over time. The hump-shaped curve displayed by species diversity (i.e., species richness and Simpson diversity index) found in our study, peaking at intermediate levels of aridity, was contrary to our first hypothesis. However, similar curves to the one shown in this work were also found in other studies such as along a gradient from arid to dry subhumid climates in global drylands (Soliveres et al., 2014), an altitude gradient (Chawla et al., 2008), and across disturbance gradient (Wilkinson, 1999).

In our case, sites with intermediate aridity levels along the spatial gradient can be interpreted as an ecotone between semi-arid and more humid sites, where most species, both from drier and more mesic environments, may co-exist, still finding adequate environmental conditions to survive (Gross et al., 2000; Suding et al., 2005), at least in some sites. The co-existence of different species along this ecotone can be related to a high spatial environmental heterogeneity characteristic of tropical dry forests (Moro et al., 2014; Oliveira et al., 2020b) that can lead to a heterogeneous distribution of vegetation, leading to high niche diversity allowing multiple species to coexist (Orians, 1982; Pausas and Austin, 2001). Species dominating in more arid conditions need traits that allow them to withstand long periods of drought, as is the case of, e.g., Bromeliaceae species, which have leaves arranged in a rosette, that function as “storage tanks” of water and facilitate the acquisition of nutrients (Takahashi et al., 2007). These strategies are different from the ones dominating in more mesic sites, where we found, e.g., more evergreen trees such as *Cynophalla flexuosa* (L.) J. Presl (Capparaceae). Thus, species’ ability to persist and dominate in the plant community is a result of the environmental filtering of their traits (to deal with water and nutrient availability), and also of species interactions, e.g., their competitive ability under particular ecological conditions (competitors or stress tolerators, sensus Grime, 1977; Walker et al., 2003).

The dominance of different plant ecological strategies in the extremes of the gradient is also supported by the results of functional diversity. The tendency for higher functional diversity in drier sites, suggests that higher aridity selects for particular drought-adapted species with diverse functional traits, that allow them to avoid or tolerate those stressful conditions. An example is the coexistence of species with distinct photosynthetic pathways in drier sites, including the CAM of Cactaceae species. Again, this may be related to a higher heterogeneity in resource distribution (higher niche differentiation) in drier sites, leading to the coexistence of species with dissimilar resource acquisition strategies (Stubbs and Wilson, 2004; De Bello et al., 2006). The results also demonstrate that species richness and functional diversity are not always positively correlated (Botta-Dukát, 2005), and that a greater number of species may correspond to lower functional diversity. Yet, these findings contradict our expectations of finding a lower functional diversity in the more arid sites, as a result of environmental filtering, as was found for other (Mediterranean) drylands (e.g., Nunes et al., 2017). These contrasting results may be because our study (i) analyzed different functional traits (e.g., chemical defense exudation, photosynthetic pathways and leaves arranged in a rosette), (ii) encompassed a greater geographic coverage (ca. 700 km), and (iii) considered a wider aridity range (aridity index from 0.27 to 0.69), compared to the one performed in Mediterranean drylands.

The higher functional diversity found in drier sites, coupled with low species richness, led to a lower FR, contradicting our expectations (third hypothesis). This means that drier sites have few species with different functional traits and resource acquisition strategies among each other, to cope with high niche differentiation in a heterogeneous environment where resources are scarce, thus avoiding competing for the same resources. Hence, as aridity increases, ecosystem functioning in this tropical dry forest is largely assured by only a few species with unique functions, displaying low FR. Within this context, the loss of species with key functions, or a set of species that exhibited similar ecological functions, can compromise the stability, resistance and resilience and further increase the susceptibly (Fonseca and Ganade, 2001; Bellwood et al., 2003) of this ecosystem to changes in aridity. This statement is supported by the importance of: (i) species diversity in controlling the stability of ecosystems and communities (e.g., Ehrlich and Walker, 1998); (ii) functional diversity in improving the resistance of dryland ecosystems to aridity (e.g., Volaire et al., 2014); and (iii) species redundancy in ensuring ecosystem resilience to disturbance (e.g., McCann, 2000).

## Concluding Remarks

Our results observed along a large spatial aridity gradient are a proxy of what might happen with climate change over time and have alarming implications for the future of these drylands. Ecological indicators based on biodiversity metrics, as shown in our study, are fundamental to comprehensively describe diversity change and species loss in drylands and therefore at least these indicators should be used over time. Caatinga’s Tropical dry forests are among the most diverse drylands. Yet, despite its high plant functional diversity, our findings regarding low FR suggest a high susceptibility of this ecosystem to an increase in aridity due to climate change. In what concerns the management of Caatinga, the knowledge acquired in this work can be used as an early warning, to timely adopt strategies to improve its stability, resistance and resilience to future environmental changes. This is particularly relevant given that this ecosystem has already experienced an increase and rapid anthropic-derived degradation (Sfair et al., 2018; Ribeiro et al., 2019). Extra negative impacts due to an increase in temperature and reduced precipitation associated with climate change, may lead to the loss of species with key traits, compromising the functioning of this ecosystem. Furthermore, these negative impacts can accelerate desertification processes, which will affect 28.6 million people highly dependent on local natural resources (da Silva et al., 2018).

To sum up, the responses of complementary diversity metrics to aridity and the interdependence between them shown in this work, contribute to a better understanding of the susceptibility of this ecosystem to climate change, and may help to define strategies to improve the stability, resilience, and resistance to ongoing and future global changes in drylands.

## Data Availability Statement

The original contributions presented in this study are included in the article/Supplementary Material, further inquiries can be directed to the corresponding author.

## Author Contributions

AO: conceptualization, methodology, formal analysis, investigation, and writing. AN and CB: conceptualization, methodology, formal analysis, and writing. MO: formal analysis and writing. RR: investigation and writing. All authors contributed to the article and approved the submitted version.

## Conflict of Interest

The authors declare that the research was conducted in the absence of any commercial or financial relationships that could be construed as a potential conflict of interest.

## Publisher’s Note

All claims expressed in this article are solely those of the authors and do not necessarily represent those of their affiliated organizations, or those of the publisher, the editors and the reviewers. Any product that may be evaluated in this article, or claim that may be made by its manufacturer, is not guaranteed or endorsed by the publisher.

## Footnotes

1
http://www.hvasf.univasf.edu.br/
