# Supplementary material for: How Do Taxonomic and Functional Diversity Metrics Change Along an Aridity Gradient in a Tropical Dry Forest?
Source: Front Plant Sci. 2022 Jul 7;13:923219. doi: 10.3389/fpls.2022.923219 (PMC9302379; doi:10.3389/fpls.2022.923219)
Supplement: Supplementary file 1 [file Table_1.DOC]

**Supplementary Table A1**. Results of the models built to analyse the response of Species richness, Simpson diversity index, Functional diversity, and Functional redundancy to aridity (the aridity index). Predictor estimates, standard errors, P values and R² are presented.

| **Response Variable** | **Predictor** | **Estimate** | **Std Error** | **P value** | **Adjusted R^2^** |
| --- | --- | --- | --- | --- | --- |
| Species richness | Aridity | 11.847 | 4.667 | 0.013 | 0.06* |
|  | Aridity^2^ | -13.090 | 5.260 | 0.014 |  |
| Simpson index | Aridity | 0.307 | 0.137 | 0.027 | 0.03* |
|  | Aridity^2^ | -0.337 | 0.152 | 0.029 |  |
| Functional diversity | Aridity | -0.096 | 0.038 | 0.014 | 0.05* |
| Functional redundancy | Aridity | 0.076 | 0.024 | 0.006 | 0.06** |
